# Fast, interleaved, Look‐Locker–based *T*_1_ mapping with a variable averaging approach: Towards temperature mapping at low magnetic field

**DOI:** 10.1002/nbm.4826

**Published:** 2022-09-27

**Authors:** Marco Fiorito, Maksym Yushchenko, Davide Cicolari, Mathieu Sarracanie, Najat Salameh

**Affiliations:** ^1^ Department of Biomedical Engineering Center for Adaptable MRI Technology, University of Basel Allschwil Switzerland; ^2^ Department of Physics University of Pavia Pavia Italy

**Keywords:** hyperthermia, low‐field MRI, MR thermometry, *T*_*1*_ mapping, temperature mapping

## Abstract

Proton resonance frequency shift (PRFS) is currently the gold standard method for magnetic resonance thermometry. However, the linearity between the temperature‐dependent phase accumulation and the static magnetic field B_0_ confines its use to rather high‐field scanners. Applications such as thermal therapies could naturally benefit from lower field MRI settings through leveraging increased accessibility, a lower physical and economical footprint, and further consideration of the technical challenges associated with the integration of heating systems into conventional clinical scanners. $T_{1}$‐based thermometry has been proposed as an alternative to the gold standard; however, because of longer acquisition times, it has found clinical use solely with adipose tissue where PRFS fails. At low field, the enhanced $T_{1}$ dispersion, combined with reduced relaxation times, make $T_{1}$ mapping an appealing candidate. Here, an interleaved Look‐Locker–based $T_{1}$ mapping sequence was proposed for temperature quantification at 0.1 T. A variable averaging scheme was introduced, to maximize the signal‐to‐noise ratio throughout $T_{1}$ recovery. In calibrated samples, an average $T_{1}$ accuracy of 85% ± 4% was achieved in 10 min, compared with the 77% ± 7% obtained using a standard averaging scheme. Temperature maps between 29.0 and 41.7°C were eventually reconstructed, with a precision of 3.0 ± 1.1°C and an accuracy of 1.5 ± 1.0°C. Accounting for longer thermal treatments and less strict temperature constraints, applications such as MR‐guided mild hyperthermia treatments at low field could be envisioned.

## Graphical abstract

Through the proposed Look‐Locker–based $T_{1}$ mapping sequence, temperature maps of homogeneous doped‐water solutions at 0.1 T could be obtained in 10 min. The use of a variable averaging scheme enhanced $T_{1}$ estimation accuracy, leading to a temperature precision of 3.0 ± 1.1°C and an accuracy of 1.5 ± 1.0°C. Given the prolonged thermal treatments and the less strict temperature constraints, applications in MR‐guided hyperthermia treatments at low field could be envisioned.

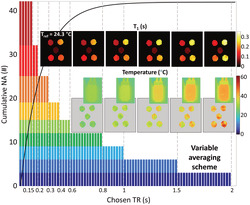

**Abbreviations used**
bSSFP: balanced steady‐state free precession
GRE: gradient‐recalled echo
IGT: image‐guided therapies
IR: inversion recovery
LL: Look‐Locker
NA
: number of signal averages
PRFS: proton resonance frequency shift
RF: radiofrequency
SR: saturation recovery
SNR: signal‐to‐noise ratio
s.d.: standard deviation
VFA: variable flip angle

## 1 INTRODUCTION

Image‐guided therapies (IGT) consist of using imaging modalities to aid and complement specific surgical procedures and therapeutic interventions. Providing images with unparalleled soft tissue contrast, magnetic resonance imaging (MRI) has become a major contender in IGT, because of its inherent absence of ionizing radiations and the possibility to complement anatomical images with quantitative physiological information (e.g., blood flow, diffusion/perfusion, stiffness). Over roughly three decades, interventional and intraoperative MRI have been leveraged for navigation, motion tracking during intraoperative and biopsy procedures,^1^, ^2^ and for real time or quasi‐real time feedback for MR theragnostic methods (e.g., MR‐guided high intensity focused ultrasound surgery, radiofrequency [RF] hyperthermia, radiation, and proton therapy).^3^ The continuous evolution of MRI towards higher magnetic fields also contributed to boosting the appeal of this imaging modality among clinicians, considering the higher level of detail achievable with the promise of reduced scan times.^4^ Nonetheless, such benefits come at the price of lowered compatibility with the settings and procedures typical of an operating room. Indeed, high‐field scanners require a fully MR compatible environment, from surgical instruments to vital‐sign monitoring equipment. Projectile effect and RF heat deposition caused by metallic instruments and implants are potential hazards associated with strong magnetic environments.^5^ Image distortion and signal loss resulting from susceptibility artifacts are also directly related to magnetic field strength and can hinder the interpretation of the images and cause erroneous diagnoses.^6^ Besides, the limited bore size typical of conventional high‐field scanners makes them less suitable for bedside interventions compared with the open geometry of many low‐field designs.^7^, ^8^ The recently revived interest in low‐field MRI^9^, ^10^, ^11^, ^12^, ^13^, ^14^, ^15^ offers opportunities to tackle the aforementioned problems, with the potential to fit challenging environments such as intensive care units,^16^ while still providing clinically relevant information.^17^, ^18^

Thermal medicine is counted among the applications that largely benefit from MR guidance. Leveraging the dependence of tissue magnetic properties with temperature, MRI currently provides the only noninvasive option for temperature mapping.^19^ MR thermometry most commonly relies on proton resonance frequency shift (PRFS),^20^ that is, the phenomenon occurring when water molecules are exposed to higher or lower temperatures than the reference body temperature, leading to a temperature‐dependent shift in their precession frequency.^21^ The change in accumulated phase scales linearly with the temperature variation, with a sensitivity of ~0.01 ppm/°C in aqueous tissue.^22^, ^23^ PRFS‐based thermometry was successfully employed in several clinical cases and for a range of thermal therapies.^20^ Nevertheless, the negligible temperature sensitivity measured in adipose tissue restricts its applicability to aqueous tissue. Additionally, its employment is limited to conventional high‐field scanners (i.e., 1.5 and 3 T), because of the linear dependence of the accumulated phase shift on the magnetic field strength $B_{0}$. Because of technical limitations and the high costs associated with the purchase and maintenance of high‐field scanners, today the use of temperature probes is still the preferred method for temperature mapping.^24^ Alternative methods that can provide increased flexibility are gaining interest, based on the mapping of, among others, spin–lattice relaxation time $T_{1}$, spin–spin relaxation time $T_{2}$, and molecular diffusion.^25^ These methods exploit the dependence of certain parameters on temperature over an investigated temperature range. Among them, $T_{1}$ has the potential to arise as the parameter of reference at low magnetic field by virtue of a higher dispersion^26^, ^27^, ^28^ and an increased temperature sensitivity^29^ compared with what is reported for high‐field strengths. A nearly linear trend was measured both in water‐ and fat‐based samples between $T_{1}$ and temperatures ranging from room temperature to 43°C,^30^, ^31^, ^32^ the latter being conventionally considered the index temperature associated with thermal‐induced cytotoxicity and used as a reference for thermal iso‐effective dose calculations in hyperthermia applications.^33^, ^34^ These properties make $T_{1}$‐based MR‐guided hyperthermia extremely attractive at low magnetic field. Yet the employment of $T_{1}$‐based thermometry is not limited to low‐field applications, and the possibility to measure temperature in adipose tissue has been successfully exploited in hybrid PRFS/ $T_{1}$ techniques performed with high‐field clinical scanners.^35^, ^36^ Initially relying on the variation in signal magnitude of $T_{1}$‐weighted images with temperature,^32^ this approach was gradually abandoned in favor of quantitative techniques. Several sequences for local $T_{1}$ quantification have been developed over the years; while inversion recovery (IR) can be considered the gold standard sequence for $T_{1}$ quantification,^37^ variable flip angle (VFA)^36^ and Look‐Locker (LL) methods^38^ are often preferred because of their shorter acquisition times. Having found wide employment in cardiac MRI at clinical fields (1.5 and 3 T),^39^, ^40^, ^41^ the development of fast and robust $T_{1}$ mapping sequences at low fields is an ongoing field of research.

In this paper, an LL‐based partial saturation recovery (SR) sequence with interleaved slice acquisition was implemented for temperature mapping at low field (0.1 T), based on the work of Deichmann.^42^ To compensate for the inherent low signal‐to‐noise ratio (SNR) of low fields, the proposed work embeds a variable averaging approach in the transient state of the proton recovery process. In addition, temperature maps could be successfully derived from $T_{1}$ maps in calibrated doped‐water vials, without leveraging prior knowledge of their respective $T_{1}$ sensitivity with temperature.

## 2 THEORY

### 2.1 $T_{1}$ mapping

To capture $T_{1}$ relaxation and assess its dependence on temperature, a LL‐based sequence was employed. The standard LL approach used for IR involves the application of an initial 180° RF pulse, followed by a train of sequential low‐angle pulses α, equally spaced by a chosen sampling time ( ${\mathrm{TR}}_{\alpha }$).^43^ Each of these low‐angle pulses allows measuring a fraction of the magnetization vector $M_{z}$ at the corresponding ${\mathrm{TR}}_{\alpha }$. The time‐dependent recovery of the remaining net magnetization is then described by the parameter $T_{1}^{*}$, which represents an underestimation of $T_{1}$ affected by α and by the chosen ${\mathrm{TR}}_{\alpha }$, as described in Equation (1):

(1) $$\frac{1}{T_{1}^{*}}=\frac{1}{T_{1}}-\frac{\ln\left(\cos\;\alpha \right)}{{\mathrm{TR}}_{\alpha }}.$$

After a time $t\geq 5\;T_{1}$, the magnetization vector converges to an asymptotic value $M_{0}^{*},$ which relates to the net magnetization $M_{0}$ through Equation (2) provided that $\mathrm{TR}\ll T_{1}^{*}$,

(2) $$M_{0}^{*}=M_{0}\frac{T_{1}^{*}}{T_{1}}.$$

Finally, a delay is added to let the magnetization fully recover and reach its value at thermal equilibrium $M_{0}$.

In this work, a 90° saturation pulse replaces the initial 180° inversion pulse^44^ and eliminates the need for full recovery of the net magnetization, hence significantly accelerating the acquisition. This is referred to as a partial SR sequence^45^ and the corresponding evolution of the magnetization vector $M_{z}$ with time follows Equation (3).^42^

(3) Mzt=M0**1−e−tT1*.

$M_{0}^{**}$ represents the new value assumed by the longitudinal magnetization at the end of each TR and reads:

(4) M0**=M0*1−e−TRT1*.

**FIGURE 1:**
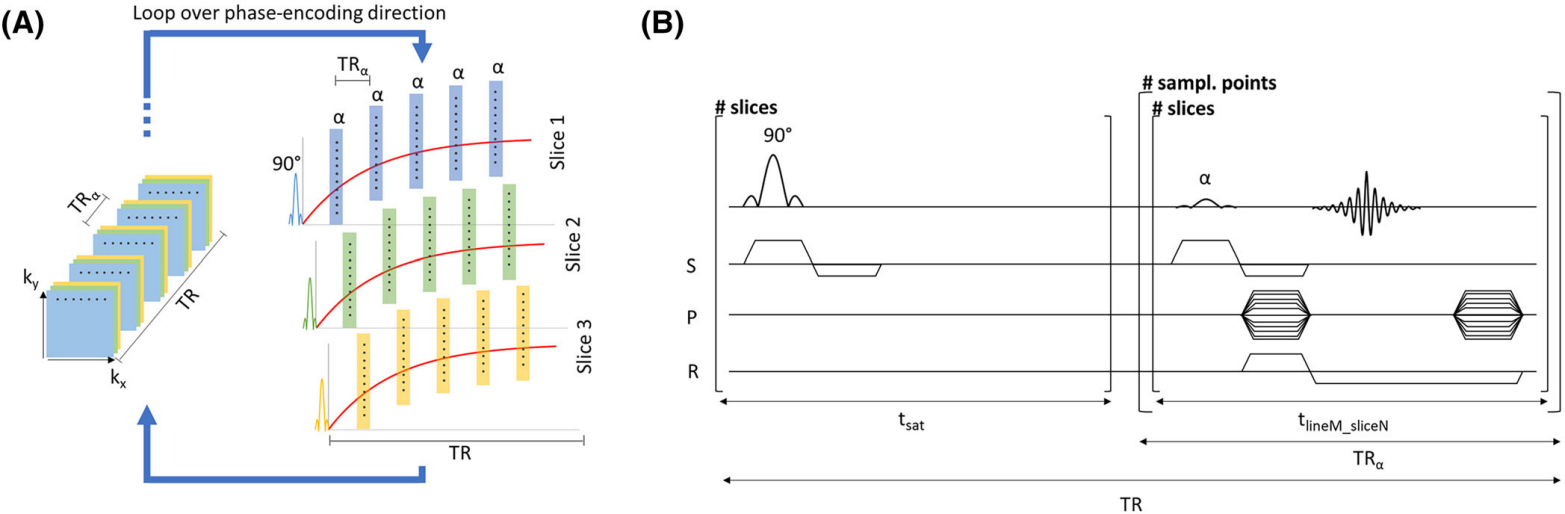
Schematics of the LL sequence. (A) The interleaved architecture allows to sample the $T_{1}^{*}$ recovery for different slices without increasing TR. After each TR, the acquisition is repeated for the next k‐space line. (B) GRE sequence diagram. S, P, and R refer to the slice, phase, and frequency encoding direction. $t_{\text{lineM}\_\text{sliceN}}$ indicates the time required to acquire a single line M for a single slice N of *k*‐space at a specific sampling point. $t_{sat}$ is the time dedicated to the 90° excitation of a single slice N and here it was set to be equivalent to $t_{\text{lineM}\_\text{sliceN}}$. $TR_{\alpha }$ is the time necessary to acquire the same line of *k*‐space for all encoded slices and also represents the time delay between the acquisition of the same line of *k*‐space at two consecutive time points. GRE, gradient‐recalled echo; LL, Look‐Locker

Because of the use of saturation pulses, retrieval of $T_{1}$ cannot be performed from Equation (2), as an estimate of $M_{0}$ cannot be obtained. However, one can convert the estimated $T_{1}^{*}$ into $T_{1}$ using Equation (1), provided there is pixel‐wise knowledge of the small flip angle α. To further accelerate the sequence, an interleaved multislice scheme was considered. In this case, a train of slice‐selective saturation pulses, spaced by $t_{\mathrm{sat}}\leq {\mathrm{TR}}_{\alpha }$, was used to consecutively excite adjacent slices. In this way, interleaved slice‐selective α pulses allow to collect data from one slice while the spins contained in the others are relaxing. Assuming Cartesian *k*‐space sampling, the number of phase‐encoding steps, the number of signal averages ( NA), and TR become the main contributors to the sequence’s total acquisition time. A schematic of the interleaved sequence is presented in Figure 1A,B. It is relevant to notice that the number of slices does not impact the acquisition time and can be increased at the price of a sparser sampling of the relaxation curve over the same time. The maximum number of slices is determined by:

(5) $$N_{\mathrm{sl}}^{\max}\leq \frac{{\mathrm{TR}}_{\alpha }}{t_{\text{lineM}\_\text{sliceN}}},$$

where $t_{\text{lineM}\_\text{sliceN}}$ is the time required to tip the magnetization vector by an angle α and to acquire one *k*‐space line *M* of one slice *N*. A different ${\mathrm{TR}}_{\alpha }$, however, will affect the resulting $T_{1}^{*}$ (see Equation 1) and must be carefully chosen.

### 2.2 Variable averaging

**FIGURE 2:**
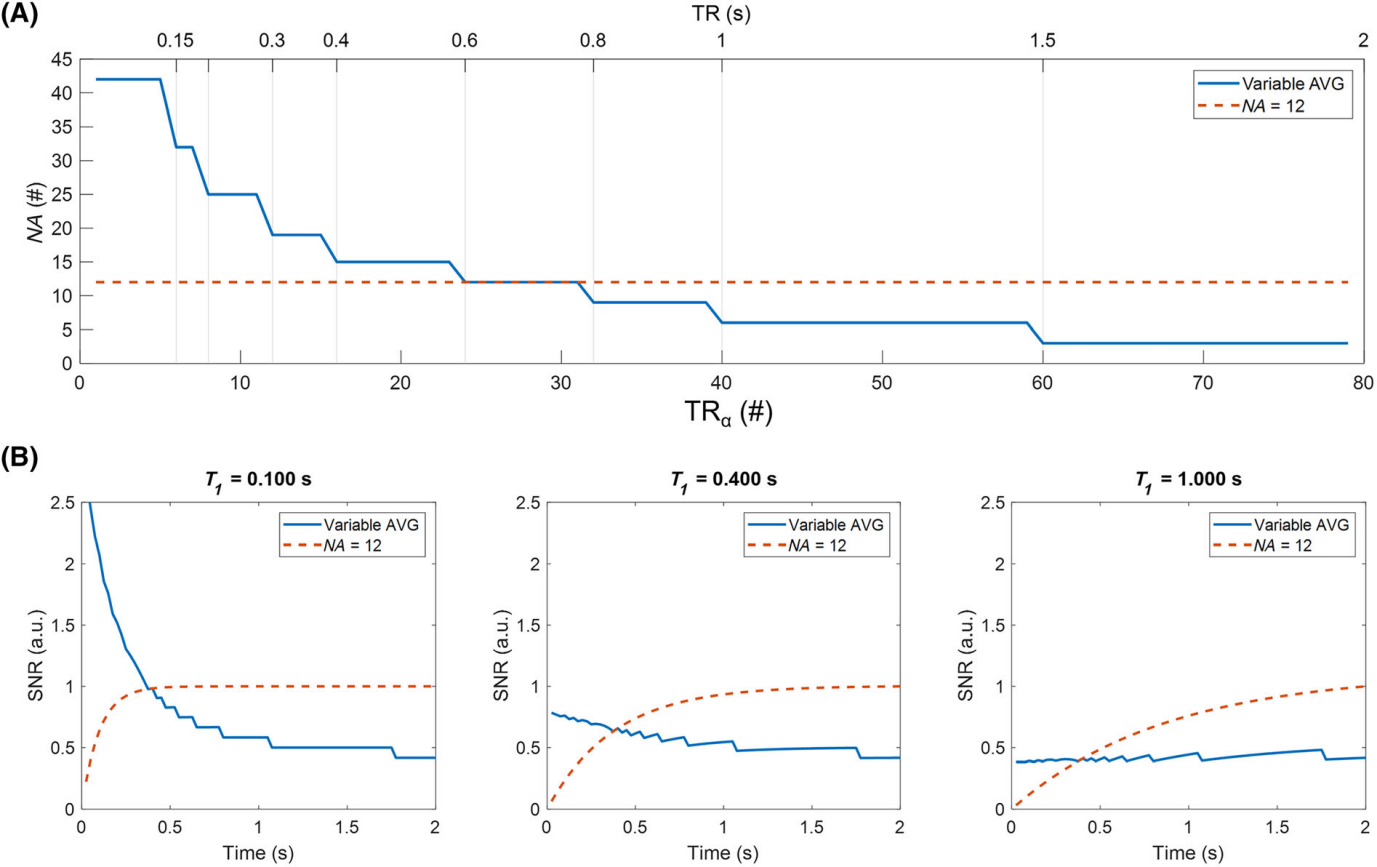
Schematic representation of the variable averaging method. (A) Averaging scheme employed for the experiments (blue) and constant averaging scheme yielding the same total acquisition time (orange). Through the variable averaging scheme, thanks to the shorter TR, the acquisition of the images at the shortest sampling points could be repeated a higher number of times. (B) Simulated signal‐to‐noise ratio (SNR) of the variable averaging scenario (blue) versus constant averaging (orange) over magnetization recovery time for different $T_{1}$ values. The variable NA compensates for the intrinsic lack of signal of the transient state of the relaxation process, yielding increased SNR. AVG, averaging; NA, number of signal averages

The need for multiple averaging is a common requirement of low‐field scanners because of low SNR, which is seldom necessary at high fields. In the current study, variable averaging is proposed, which aims to increase SNR in the early phase of the captured magnetization recovery, intrinsically characterized by a lower signal and a steep slope. In essence, unlike conventional LL approaches, full magnetization recovery is not required between two saturation pulses selecting the same slice.^46^ Hence, a shorter TR can be employed every time the same set of 2D images is reacquired. This in turn reduces the length of the train of α‐pulses, leading to the sampling of only a part of the recovery curve. The saved acquisition time can then be reinvested to further sample the early stages of the $T_{1}^{*}$ recovery, where the signal is intrinsically weak. Through the proposed method, a higher NA in the most sensitive phase of the recovery curve is expected to improve the overall fitting outcome. In Figure 2A, the variable averaging scheme used in this study is compared with a standard acquisition leading to the same scan time. The simulations presented in Figure 2B show how the SNR obtained from the proposed method does not follow the relaxation process any more; instead, the signal from the parts of the curves characterized by a steeper growth is maximized.

### 2.3 Temperature mapping

Over the range of temperatures typically employed for hyperthermia treatments, $T_{1}$ dependence on the absolute temperature T can be modeled through an exponential function, as the one given in Equation (6)^30^, ^31^:

(6) $$T_{1}\propto e^{-\frac{E}{k_{B}T}}.$$

Here, $k_{B}$ is the Boltzman's constant and E defines the activation energy of the relaxation process. For the limited temperature range typically used in mild hyperthermia, Equation (6) can be approximated with

(7) $$T_{1,i}=k_{i}T+c_{i},$$

where $k_{i}$ defines the temperature sensitivity and is specific for the investigated tissue or sample, as indicated by the subscript i. $T_{1}$ cannot be directly converted into an absolute temperature value. This is possible only if the temperature of a reference $T_{1}$ map is known. Alternatively, the difference in temperature ΔT associated with the variation $\Delta T_{1}$ can be quantified. Naming $T_{1}^{a}$ the value measured at a reference temperature $T^{a}$, and $T_{1}^{b}$ the corresponding measure at $T^{b}$, one can relate these quantities through Equation (8):

(8) $$T^{b}-T^{a}=\frac{1}{k_{i}}\left(T_{1,i}^{b}-T_{1,i}^{a}\right).$$

The sensitivity must be calculated a priori from a sample calibration or extracted from the values reported in the literature. In the proposed work, the $k_{i}s$ of 15 aqueous solutions were found to follow a linear dependence on $T_{1}$ at a reference temperature $T^{a}$. A linear interpolation can then be used to express the sensitivity k as a function of $T_{1}^{a}$ through a new parameter K:

(9) $$k=KT_{1}^{a}.$$

Replacing Equation (9) in Equation (8) yields:

(10) $$T^{b}-T^{a}=\frac{1}{K}\left(\frac{T_{1}^{b}}{T_{1}^{a}}-1\right).$$

Unlike in Equation (8), converting $T_{1}$ variation into a temperature change can now be performed from sole knowledge of the parameter K, the calculation of which does not require prior knowledge of the investigated water samples.

## 3 MATERIALS AND METHODS

### 3.1 Sample preparation and calibration

To investigate the impact of temperature on $T_{1}$, 15 glass vials containing different concentrations of ${\text{MnCl}}_{2}$ dissolved in deionized water were prepared, as reported in Table 1. Ground truth assessment of the dependence of $T_{1}$ on temperature was performed by placing all samples in a temperature‐controlled water circulating system (PD7LR‐20, PolyScience AG, Switzerland), employed for this experiment as a water bath. The temperature was progressively increased from 25 to 60°C and then decreased back to 25°C, in steps of 5°C. Each temperature point was maintained for 10 min to ensure thermal equilibration prior to any calibration measurement. For each step, the vials were sequentially taken out and quickly positioned in a 5‐cm bore, 0.1‐T biplanar scanner (Bouhnik S.A.S., France), where a conventional spectroscopic SR sequence was used for bulk $T_{1}$ measurements. Logarithmically spaced sampling was preferred to achieve better coverage of the steepest part of the recovery curve, and further reduce the sequence time. The total acquisition time for each of the first eight samples was 47 s, 62 s for samples 9 to 12, and 74 s for the remaining three. A few seconds prior to the acquisitions, the temperature of each sample was recorded outside the bath using a thermal camera (Testo 865, Testo SE & Co. KGaA, Germany) with an accuracy of ±2°C.

**TABLE 1:** Linear parameters describing the temperature sensitivity of $T_{1}$ of the 15 samples

Sample	[ ${\text{MnCl}}_{2}$] (μM)	Slope (ms/°C)	Intercept (ms)	r^2^
S1	1,350	1.25 ± 0.08	21 ± 3	0.95
S2	650	2.55 ± 0.07	37 ± 3	0.99
S3	450	3.7 ± 0.1	48 ± 5	0.99
S4	300	5.2 ± 0.2	74 ± 6	0.99
S5	250	6.4 ± 0.2	80 ± 8	0.99
S6	200	7.5 ± 0.2	107 ± 8	0.99
S7	160	9.5 ± 0.3	111 ± 10	0.99
S8	150	10.5 ± 0.3	131 ± 13	0.99
S9	120	14.4 ± 0.6	122 ± 24	0.98
S10	100	14.1 ± 0.6	183 ± 24	0.98
S11	80	17.0 ± 0.8	185 ± 32	0.97
S12	70	20.1 ± 0.8	195 ± 30	0.98
S13	60	23 ± 1	268 ± 39	0.98
S14	40	30 ± 2	272 ± 68	0.96
S15	10	49 ± 3	574 ± 102	0.96

### 3.2 Temperature mapping experiment

**FIGURE 3:**
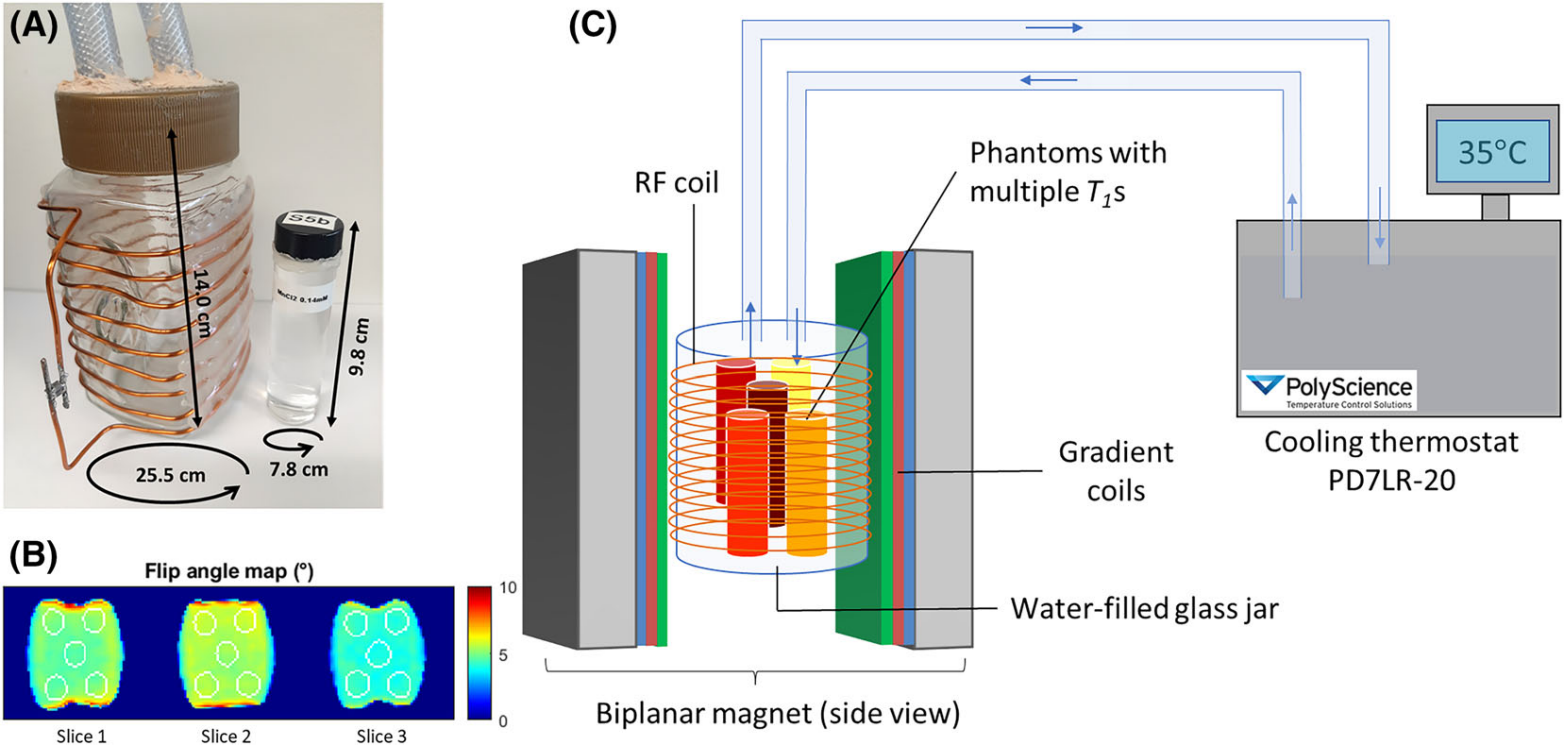
Experimental setup. (A) 15 ${\text{MnCl}}_{2}$‐doped water samples (outer circumference × height ≈ 7.8 × 9.8 cm) were created and inserted (in groups of five) into a glass jar (outer circumference × height ≈ 25.5 × 14 cm). A RF coil was wound around the jar for both transmit and receive operations. (B) The flip angle map of the coil is presented, obtained using the low angle method and a nominal flip angle of 5°. The white contours highlight the position of the vials in the three slices. (C) The water‐filled glass jar, positioned at the center of a 0.1‐T biplanar magnet, was connected to a temperature‐controlled water circulating system. The temperature was increased stepwise and $T_{1}^{*}$ maps were acquired after 10 min of thermal stabilization (five samples at a time)

The characterized samples were completely immersed in a water‐filled glass jar (Figure 3A) placed in an 18‐cm bore, resistive 0.1‐T biplanar scanner (EAR54L, Drusch & Cie, France). A custom solenoid RF coil was wound around the jar (nine turns), used for both transmit and receive operations through a passive transcoupler (NMR service, Germany). RF transmit operations were performed via a 500‐W pulsed amplifier (BT00500‐AlphaS, Tomco Technologies, Australia). For receive operations, the solenoid RF coil was connected to a custom‐made preamplifier.^47^ To set and maintain the temperature of the samples, the temperature‐controlled water circulating system was connected to the jar outlets. $T_{1}^{*}$ maps of all vials, subdivided in groups of five, were initially acquired at the reference temperature of 25°C. This first part of the experiment aimed to define the intrinsic limits of the proposed sequence to correctly characterize a wide range of $T_{1}s$. A schematic representation of the experimental setup is displayed in Figure 3C.

In a second phase, samples S1 to S5 only were repositioned in the scanner and the temperature was successively set to 30, 35, 40, 43, and 45°C. The choice to focus on the first five samples was dictated by the shorter $T_{1}$ expected in soft tissues at the employed field strength.^29^ Similar to the calibration experiments, a 10‐min settling time was used for thermal equilibration prior to scanning. A $T_{1}^{*}$ map was acquired for each temperature point. To account for heat dissipation between the heated water tank and the samples, the thermal camera was used to monitor the temperature of the whole jar. These measurements provided the ground truth values for the MRI‐based temperature estimation.

### 3.3 Imaging protocol

**FIGURE 4:**
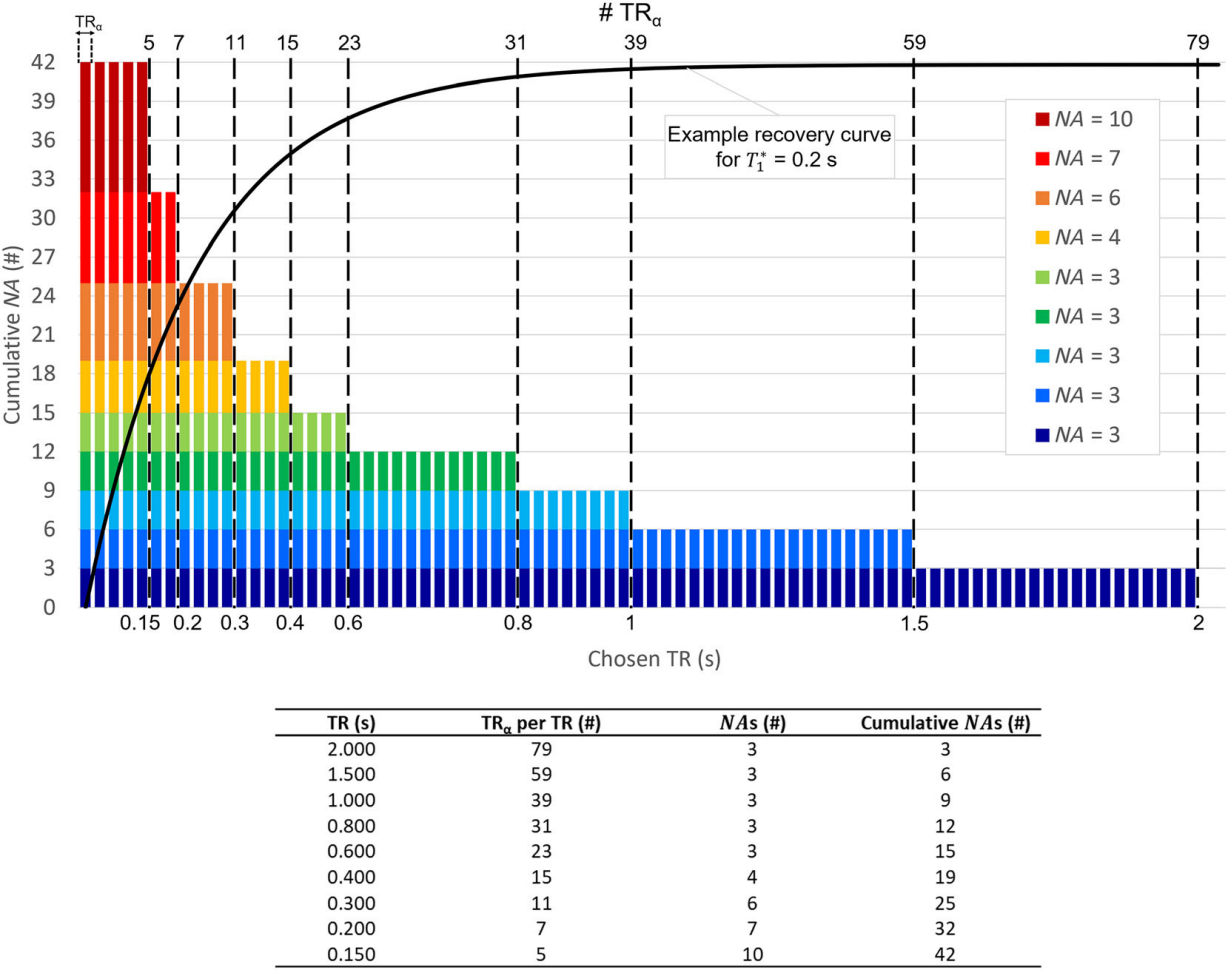
Employed variable averaging scheme. (Top) Visual representation of the employed variable averaging process. Each acquisition, characterized by a specific TR, is repeated a predefined number of times before changing TR to a shorter value. As a result, the $TR_{\alpha }$ values associated with the first part of the curve are more frequently sampled (higher NA). (Bottom) Table reporting the details of the variable averaging scheme. NA, number of signal averages

Based on the presented theory, a LL‐based interleaved sequence with partial SR was used for $T_{1}^{*}$ mapping of the prepared samples. A FLASH 2D sequence (Figure 1) was employed for the acquisition of 64 × 35‐pixel slices, sampling 24 of the 35 *k*‐space lines (i.e., ~ 70%) in the phase‐encoding direction. To that end, an undersampling mask was generated following a Gaussian probability distribution centered on the central line in *k*‐space.^48^ Sinc‐shaped slice‐selective RF pulses (600 μs) with two lobes at each end of the zero‐crossing point were used for both saturation and α pulses, with nominal α= 5°. Three adjacent slices could be acquired using a ${\mathrm{TR}}_{\alpha }$ of 25 ms. For the variable averaging strategy employed, a TE of 4 ms and a maximum TR of 2000 ms were used, leading to a maximum of 79 sampling time points per TR. The averaging scheme presented in Figure 4 was followed, resulting in a maximum NA of 42 for the first five sampling points and a minimum NA of 3 for the last 20. The scheme was chosen such that the overall sum of the number of averages for all sampling points matched that employed for a standard averaging scheme when using 12 averages (i.e., 12 averages × 79 sampling points = 948 averages). The distribution of the averages throughout the 79 time points was arbitrarily chosen to promote the SNR of the first time points, and no specific optimization strategy was employed. A total acquisition time of 10 min for a set of 237 2D images at each temperature point was achieved. For comparison, a regular averaging scheme was also employed at the reference temperature. In this case, NA = 12 and TR = 2000 ms were chosen, which yield the same acquisition time. In both cases, the in‐plane resolution was 3 × 3 mm^2^ (bandwidth ≈ 323 Hz/pixel), with a slice thickness of 20 mm.

To allow $T_{1}^{*}$ conversion to $T_{1}$, a reference $B_{1}^{+}$ map was acquired with the same spatial resolution following the low angle mapping method presented in.^49^ The spatial flip angle distribution obtained for a nominal flip angle of 5° is shown in Figure 3B (mean ± s.d. = 4.8 ± 0.6°). All sequences were developed on a Cameleon 3 MRI console (RS^2^D, France).

### 3.4 Data processing

#### 3.4.1 $T_{1}$ mapping

The following two‐parameter exponential model was used to fit the spectroscopic data:

(11) Mzt=M01−e−tT1.

After assessing the homogeneity of the variances through a Levene's test, an ANCOVA test was used to check for the absence of difference between the $T_{1}$ values obtained from the heating and the cooling process. The data were then merged into a unique dataset. A linear fit in the form y=Ax+B was employed to probe the $T_{1}$ dependence on temperature. Equation (9) was then used to fit the estimated temperature sensitivity as a function of the $T_{1}$ measured at the reference temperature of 25°C. Pearson's correlation coefficient $r^{2}$ was chosen to assess the linearity of all trends.

From the imaging experiment, a full stack of images representing the temporal evolution of the signal during magnetization recovery was obtained for each of the three slices, and at each temperature point. Each acquired *k*‐space was denoised through a 2D Hamming filter and zero‐filled to reshape the *k*‐space matrix to 128 × 69 pixels. The images obtained via 2D Fourier Transform of the acquired data were normalized by NA to account for variable averaging. An intensity‐based region‐growing algorithm was employed to segment the vials in the magnitude MR images. A pixel‐wise fit was then applied to each stack, based on the two‐parameter model of Equation (3), where $M_{0}^{**}$ and $T_{1}^{*}$ are the two free parameters and $M_{z}$ represents the pixel intensity; 95% limits of agreement were computed for the fitting parameters. To correctly scale the dynamic range between 0 and $M_{0}^{**}$ (assuming the intensity measured at the first sampling point approaches zero), the same background region was selected at each time point on the magnitude images, the mean value of which was subtracted from the corresponding image. The same process was repeated for each average as, in the case of the variable averaging approach, averaging of different images corresponding to the same time point was not performed directly during the acquisition, but in postprocessing. This was especially important for the variable averaging case, where the noise level changes throughout the sampling points. Given the nonlinear contribution from noise to the true signal in a magnitude image, a simple subtraction of the noise‐induced bias may be suboptimal, especially when the SNR is particularly low. Different methods to handle the data can be found in the literature,^50^, ^51^ although they were not used in this study.

Given that $T_{1}$ cannot be negative (i.e., $\frac{-\ln\left(\cos\alpha \right)}{{\mathrm{TR}}_{\alpha }}<\frac{1}{T_{1}^{*}}$ in Equation 1), a maximum value for the parameter $T_{1}^{*}$ was set as a boundary condition for the fitting process. Provided prior knowledge of the flip angle map, such a value can be found by calculating the limit of Equation (1) for $T_{1}\to \infty$, which now reads:

(12) $$T_{1_{\max}}^{*}=-\frac{{\mathrm{TR}}_{\alpha }}{\ln\left(\cos\alpha \right)}.$$

$T_{1}$ was calculated from $T_{1}^{*}$ by replacing the flip angle map in Equation (1). The same filtering and zero‐filling as described above were applied before obtaining the single‐slice images used to generate the flip angle map.

The accuracy of each $T_{1}$ map, whether acquired using NA = 12 or with the variable averaging scheme, was calculated as the ratio between the average $T_{1}$ estimated for each sample over all slices and the corresponding value obtained from the spectroscopic calibration curve. $T_{1}$ precision within each segmented vial was given by the average standard deviation (s.d.) normalized by the mean $T_{1}$ estimate. A Bland–Altman plot was chosen for direct comparison of the mapped $T_{1}$ values with the corresponding ground truth. A one‐sample *t*‐test was used to identify the presence of a significant bias between the compared methods. A Grubb's test was employed to detect outliers in the acquired datasets. In all statistical tests, a confidence level of 95% or higher was considered statistically significant.

#### 3.4.2 Temperature estimation

As explained in the Theory section, temperature cannot be estimated solely from $T_{1}$ maps, but requires a prior measurement of the coefficient $k_{i}$, specific for each sample. In this study, the $k_{i}$ calculated from the spectroscopic $T_{1}$ measurements were plotted against their ground truth $T_{1}$ values measured at the reference temperature. A linear fit based on Equation (9) produced an estimate of the coefficient K. Following Equation (10), this coefficient was used to generate temperature maps from the voxel‐wise ratio between each $T_{1}$ map and the one obtained at the reference temperature (i.e., 25°C). Accuracy and precision were calculated as explained in the previous section, with respect to the thermal camera measurements. Again, a Bland–Altman test was used to assess the discrepancy between the proposed MR thermometry method and the ground truth measures. The latter were calculated from the manual segmentation of the thermal camera images. For the phantom calibration, a region of interest for each vial was defined, while for the imaging experiment, the same was done for the glass jar containing five samples at each time. The mean and s.d. temperatures were then reported.

Postprocessing of all thermal camera and MRI data was carried out with MATLAB (Mathworks, R2019a), while R (RStudio, version 1.2.1335) was used for the statistical analysis.

## 4 RESULTS

### 4.1 $T_{1}$ evolution with temperature

**FIGURE 5:**
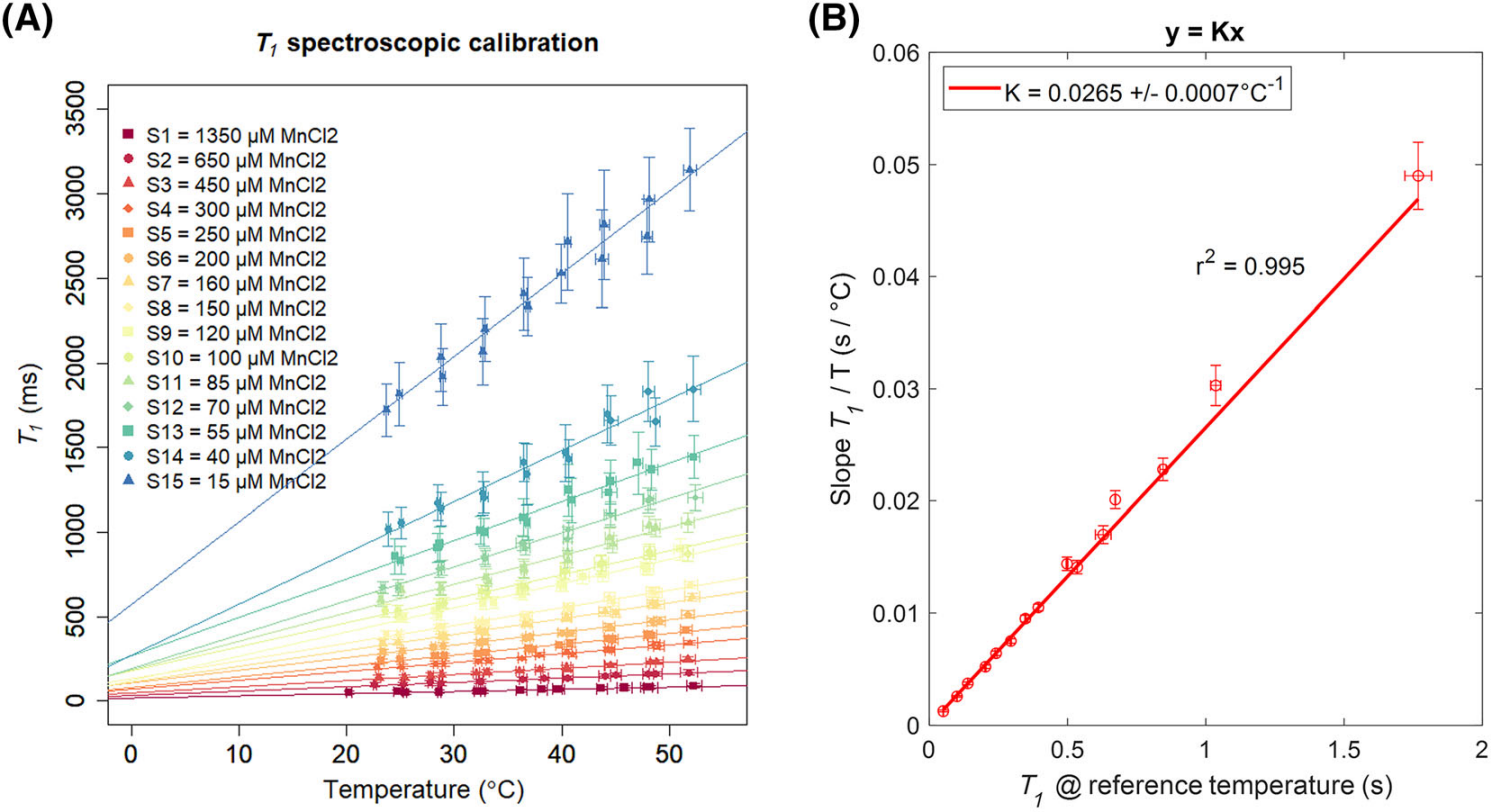
Ground truth spectroscopic $T_{1}$ calibration. (A) Each doped water sample showed a linear dependence on temperature for the investigated range of temperatures. (B) A linear trend between the temperature sensitivity of each sample and the spectroscopic $T_{1}$ value measured at the reference temperature was observed

The results obtained from the spectroscopic calibration are presented in Figure 5A. A linear fit confirmed the linear dependence between $T_{1}$ and temperature for each sample ( $r^{2}$ > 0.95). The quality of the fit and the estimated linear parameters are reported in Table 1. By plotting the estimated slopes against the mean $T_{1}$ of the corresponding samples measured at the reference temperature, a direct relationship can be observed (Figure 5B). A linear fit revealed an optimal agreement between the independent and the dependent variable ( $r^{2}$ = 0.995), as hypothesized in Equation (9). The estimated slope K was equal to 0.0265 ± 0.0007°C^−1^.

### 4.2 Accuracy and precision of the $T_{1}$ mapping sequence

**FIGURE 6:**
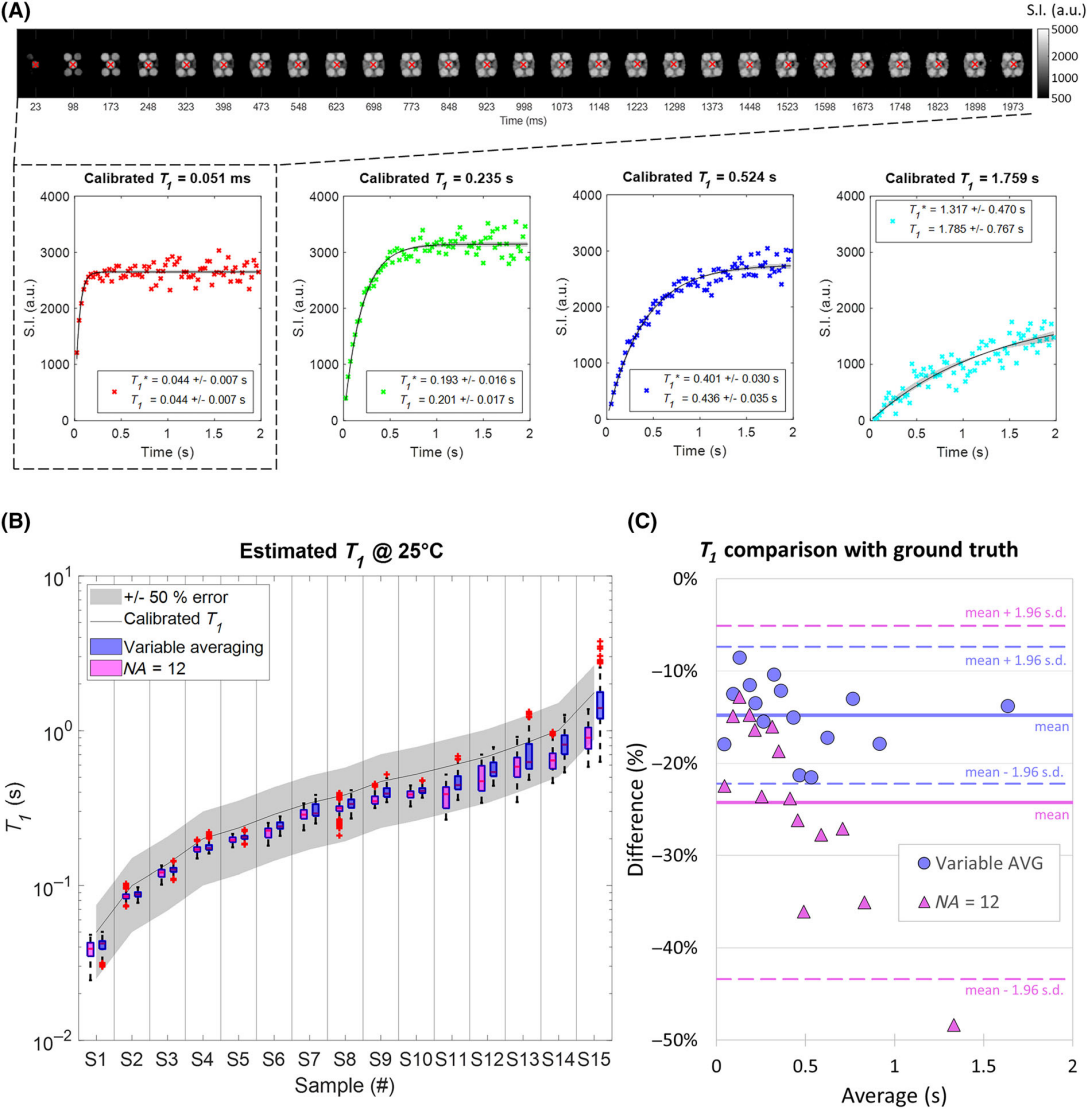
Evaluation of the $T_{1}$ mapping process at the reference temperature. (A) Top: 79 images (per slice) are acquired with variable averaging that depict the typical $T_{1}^{*}$ signal recovery in the samples (only 27 equally spaced sampled points are shown). Bottom: example of four pixel‐wise fits of the time‐dependent evolution of the measured signal intensity (S.I.) that permits $T_{1}^{*}$ and further $T_{1}$ estimation. The gray area shows the 95% confidence intervals on the retrieved parameters. (B) Boxplot comparing the $T_{1}$ distribution obtained for each sample at a nominal temperature of 25°C using the variable averaging approach (violet) and standard averaging (magenta). The line represents the ground truth values calculated from the spectroscopic calibration. The gray area gives the 50% uncertainty from the expected values. (C) Bland–Altman plot comparing the $T_{1}$ values obtained using the proposed sequence (violet) and the equivalent standard averaging method (magenta) with those retrieved through the gold standard spectroscopic saturation recovery. AVG, averaging; NA, number of averages

$T_{1}$ maps of all samples were acquired at a nominal temperature of 25°C. $T_{1}$ in the vials was estimated pixel‐wise in each slice through the fitting of the signal intensity measured at different TI, as shown in Figure 6A. Examples of the fitted curves are also presented, with the shaded areas displaying the 95% confidence intervals for the estimated parameters. The mean $T_{1}$ values calculated from each segmented sample are reported with their s.d. in the last two columns of Table 2. For comparison, the third column shows the expected values at the corresponding measured temperature, calculated using the slopes and intercepts listed in Table 1. A visual representation of the $T_{1}$ distributions can be found in Figure 6B. The line connects the calibrated $T_{1}$ values while the gray area represents a 50% error around the ground truth. One can note that variable averaging (in violet) consistently outperforms standard averaging (in magenta), being constantly closer to the calibrated $T_{1}$, especially for longer $T_{1}$. The conventional mapping sequence produced an average accuracy of 77% ± 7%, as opposed to the 85% ± 4% of the variable averaging approach. The average $T_{1}$ precision was 11% ± 6% and 10% ± 7%, respectively, although higher values were obtained for the last five samples in both cases. A comparison between the ground truth measurements and the results obtained from the mapping sequences is visible in the Bland–Altman plot in Figure 6C. A significant underestimation was observed with the variable averaging method (mean difference ± s.d. = −15% ± 4%); nonetheless the data points are well within the confidence intervals for 95% limits of agreement. A mean difference of 24% from the ground truth value was obtained for the standard averaging method, with a s.d. of 10%, caused by an increasing difficulty to accurately retrieve longer $T_{1}$ values.

**TABLE 2:** Estimated $T_{1}$ for the variable and standard averaging methods at the reference temperature, compared with the values expected from spectroscopic calibration

Sample	Measured T (°C)	Calibrated $T_{1}$ (ms)	Estimated $T_{1}$ (ms)	
			*NA* = 12	Variable averaging
S1	24.3 ± 0.2	51 ± 5	39 ± 5	41 ± 4
S2	24.3 ± 0.2	99 ± 5	85 ± 5	87 ± 4
S3	24.3 ± 0.2	138 ± 8	120 ± 7	126 ± 7
S4	24.3 ± 0.2	200 ± 10	171 ± 9	177 ± 11
S5	24.3 ± 0.2	235 ± 13	197 ± 9	203 ± 8
S6	24.2 ± 0.1	288 ± 13	220 ± 18	243 ± 15
S7	24.2 ± 0.1	341 ± 17	286 ± 28	306 ± 34
S8	24.2 ± 0.1	385 ± 20	314 ± 27	338 ± 27
S9	24.2 ± 0.1	470 ± 39	358 ± 29	399 ± 33
S10	24.2 ± 0.1	524 ± 47	387 ± 23	412 ± 21
S11	24.2 ± 0.2	596 ± 49	382 ± 65	468 ± 73
S12	24.2 ± 0.2	682 ± 58	493 ± 97	565 ± 78
S13	24.2 ± 0.2	819 ± 92	598 ± 123	713 ± 198
S14	24.2 ± 0.2	1,005 ± 145	653 ± 108	826 ± 160
S15	24.2 ± 0.2	1759 ± 261	908 ± 176	1,516 ± 500

### 4.3 $T_{1}$ maps at increasing temperature

**FIGURE 7:**
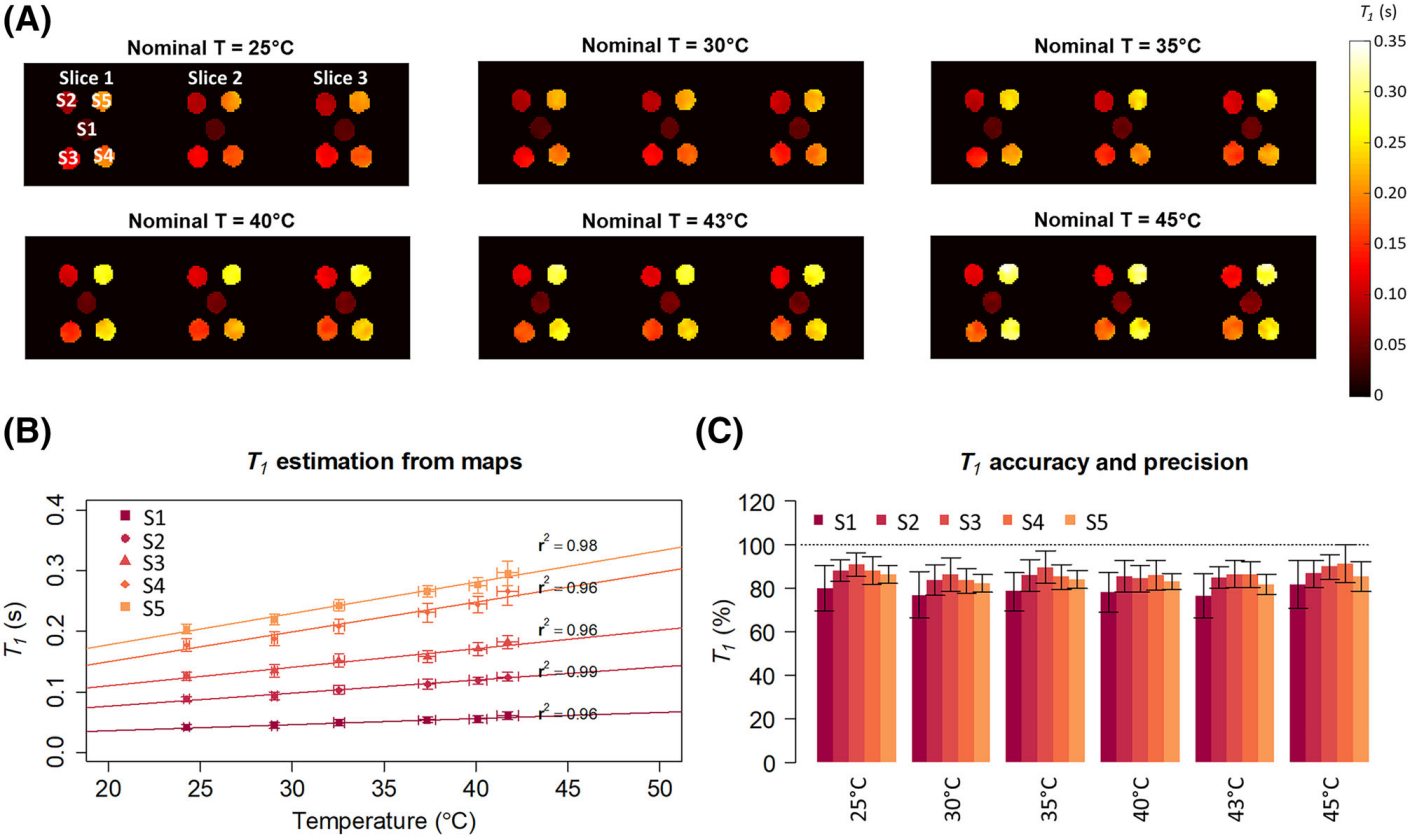
$T_{1}$ mapping at increasing temperatures. (A) $T_{1}$ maps in samples S1 to S5 measured for six temperature points. (B) Linear dependence between the estimated $T_{1}$ values (mean ± s.d. for each sample) and the measured temperatures (mean ± s.d. over the segmented glass jar). (C) Mean accuracy of $T_{1}$ estimated for each sample at each nominal temperature point. The error bars show the precision for each vial. s.d., standard deviation

The inferred $T_{1}$ maps in samples S1 to S5, obtained at six chosen temperature points (including the reference at 25°C) with variable averaging, are presented in Figure 7A. The mean $T_{1}$ calculated for each segmented sample are plotted in Figure 7B. On the x‐axis, the temperatures measured with the thermal camera are reported. Pearson's coefficients constantly above 0.95 confirm a linear trend. Figure 7C shows the calculated $T_{1}$ accuracy and precision.

The average $T_{1}$ values for each sample are also reported in Table 3. Overall, the average accuracy is 85% ± 3%, with an average $T_{1}$ precision of 7% ± 2%. Throughout the temperature points, a less accurate prediction was achieved for sample 1 (mean difference ± s.d. = 81% ± 2%); nevertheless, a Grubb's test found no significant difference associated with a specific sample or temperature.

**TABLE 3:** Average $T_{1}$ and standard deviation at different temperatures

	Measured temperature (°C)					
	24.3 ± 0.2	29.0 ± 0.2	32.6 ± 0.3	37.4 ± 0.4	40.1 ± 0.5	41.7 ± 0.6
	**Estimated** $T_{1}$ **(ms)**					
S1	41 ± 4	44 ± 5	48 ± 4	53 ± 5	54 ± 6	60 ± 7
S2	87 ± 4	93 ± 7	103 ± 7	113 ± 8	118 ± 6	124 ± 8
S3	126 ± 7	134 ± 10	151 ± 11	158 ± 10	170 ± 10	182 ± 10
S4	177 ± 11	188 ± 11	208 ± 12	231 ± 16	244 ± 14	266 ± 23
S5	203 ± 8	219 ± 9	243 ± 10	266 ± 10	276 ± 13	297 ± 20

### 4.4 Temperature maps

**FIGURE 8:**
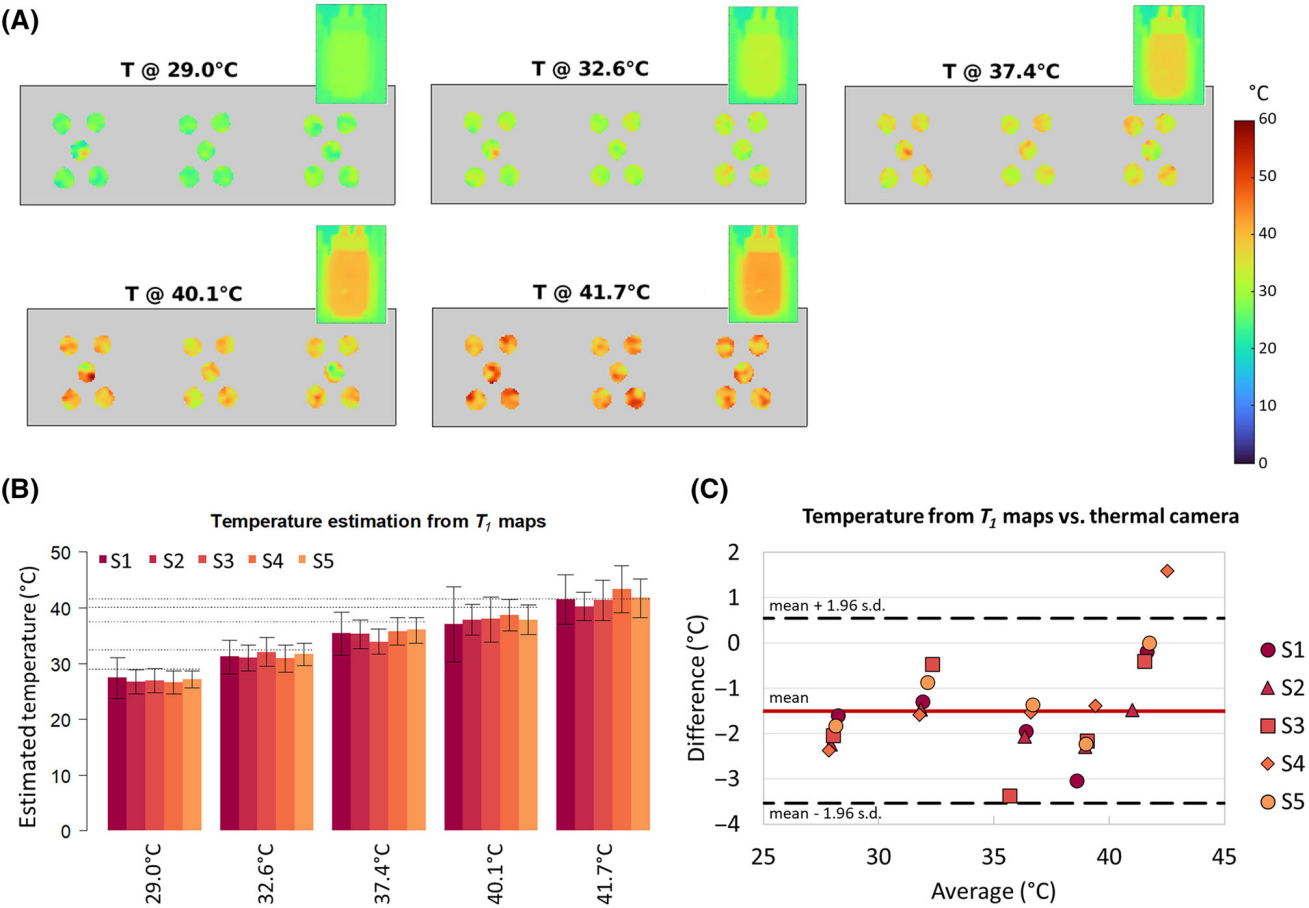
Pixel‐wise temperature estimation from $T_{1}$ maps. (A) Temperature maps from the five investigated samples and corresponding reference temperature images measured on the experimental setup (Figure 3) with the thermal camera (top‐right corner). (B) Average retrieved temperature and standard deviation (s.d.) calculated from the map of each sample at each temperature point. (C) Bland–Altman plot comparing the $T_{1}$‐based method for temperature estimation with the thermal camera measurements. s.d., standard deviation

Following Equation (10), $T_{1}$ conversion into temperature can be performed using the coefficient K calculated from the spectroscopic calibration of the samples. The generated temperature maps are shown in Figure 8A, while the quantified mean temperatures are plotted in Figure 8B and listed in Table 4. The estimated temperature difference from the values measured with the thermal camera is consistent throughout all samples and at each temperature point. The Bland–Altman plot in Figure 8C suggests a significant regular underestimation by 1.5 ± 1.0°C. The plot shows that almost all the retrieved average temperatures fall within the 95% limits of agreement between the proposed method and the ground truth measures. Only the values calculated for sample 4 at the nominal temperature of 45°C are more than 1.96 s.d.s above the mean difference. Overall, an average precision of 3.0 ± 1.1°C could be achieved. However, according to the Grubb's test, a significantly lower precision was generally observed for sample 1 (mean precision ± s.d. = 4.4 ± 1.4°C, calculated throughout the temperature points) than for the other samples. This can be visually appreciated from the larger error bars in Figure 8B.

**TABLE 4:** Average temperature and standard deviation calculated from $T_{1}$ maps

	Measured temperature (°C)				
	29.0 ± 0.2	32.6 ± 0.3	37.4 ± 0.4	40.1 ± 0.5	41.7 ± 0.6
	**Estimated temperature from** $T_{1}$ **maps (°C)**				
S1	27.4 ± 3.8	31.3 ± 3.0	35.4 ± 3.9	37.1 ± 6.7	41.5 ± 4.5
S2	26.8 ± 2.2	31.1 ± 2.3	35.3 ± 2.6	37.8 ± 2.8	40.2 ± 2.5
S3	27.0 ± 2.1	32.1 ± 2.5	34.0 ± 2.2	37.9 ± 4.0	41.3 ± 3.7
S4	26.7 ± 2.1	31.0 ± 2.4	35.8 ± 2.4	38.7 ± 2.8	43.3 ± 4.2
S5	27.2 ± 1.6	31.7 ± 1.9	36.0 ± 2.3	37.9 ± 2.7	41.7 ± 3.4

## 5 DISCUSSION

In this study, a fast, interleaved sequence was presented for $T_{1}$ mapping at low field (0.1 T), from which the temperature could then be successfully derived. A customized variable averaging method was developed to improve data quality, leading to more reliable curve fitting outcomes while mitigating the acquisition time. This approach delivered higher accuracy than a comparable standard averaging scheme, partially compensating the underestimation typically expected from LL‐based sequences.^52^ In the mapping experiments, a maximum TR of 2 s was employed for both the variable and the standard averaging approach. This value was dictated by the choice to focus on the accurate $T_{1}$ quantification of the first five samples as, with the exception of free water, $T_{1}$ in living tissue is known to decrease at low‐field regimes.^29^ Given the maximum $T_{1}$ expected from the calibration experiment for sample 5 at the highest investigated temperature (i.e., 368 ms at 45°C), the chosen maximum TR complies with the generally advised limit of $\mathrm{TR}\geq 5\;T_{1}$.^44^ As explained in the Theory section, partial SR allows to accelerate the acquisition by eliminating the need for full magnetization recovery between repetition times, at the price of an inherently limited dynamic range. With the same sequence parameters, a comparable level of accuracy could be achieved for all samples, covering $T_{1}$ values up to ~1750 ms, much longer than the conventional suggested limit. In perspective, a reduced maximum TR could suffice to provide an equally accurate estimation for shorter $T_{1}$ values, with the advantage of a reduced acquisition time. Leveraging the knowledge gained from the presented results, a tailored variable averaging scheme for a specific range of relaxation times is expected to yield better performances in terms of fitting quality and time consumption.

### 5.1 $T_{1}$ mapping limitations

A $T_{1}$ map could be produced in 10 min. Thanks to the use of saturation pulses, shorter TRs, and an undersampling scheme, the same NA = 1 would result in an acquisition time of 50 s for one dataset. Nonetheless, in this study a higher number of averages was chosen in an attempt to minimize the dispersion in the reconstructed $T_{1}$ values within each sample, which was found to be strongly impacted by SNR. An alternative acquisition strategy based on a balanced steady‐state free precession (bSSFP) readout could help in that sense, by virtue of an intrinsically higher SNR per unit time.^53^, ^54^ A 3D acquisition scheme could also be beneficial, although it would require redesigning the architecture of the proposed sequence. Besides, 2D spatially selective RF pulses are particularly widespread for local thermal therapies, because of their reduced field of view (FOV) centered around the heating focus.^55^, ^56^ Here, three adjacent slices of 20 mm sufficed to cover the whole phantom. While for clinical applications the slice thickness could be reduced, a higher number of slices would be required to cover the same FOV. Because of the interleaved architecture of the sequence, this would lead to a coarser sampling of the $T_{1}^{*}$ recovery curve, potentially hindering the correct quantification of very short relaxation times. A virtual lower limit for accurate $T_{1}^{*}$, and hence $T_{1}$ estimation, would then be directly dependent on the chosen number of slices, $N_{\text{slice}}$. In this study, a slightly higher underestimation, although not significant, was found for the shortest investigated $T_{1}$ of ~50 ms compared with the other samples. While no magnetization transfer was expected in the MnCl_2_‐doped samples used in this study,^57^ the use of off‐resonance slice‐selective RF pulses can lead to incidental signal reduction in the neighboring slices in vivo. A careful choice of the sequence parameters (e.g., longer sampling time ${\mathrm{TR}}_{\alpha },$ ^58^ lower number of slices^59^, ^60^) can alleviate its impact on $T_{1}$ estimation. Additionally, magnetization transfer is highly field‐dependent and is less pronounced at low magnetic fields.^61^ Under those circumstances, we considered that incidental magnetization transfer could be neglected, although further verification would be required.

Transmit coil's $B_{1}$ inhomogeneities produce different $T_{1}^{*}$ values from the same $T_{1}$. The employed low angle mapping method was indicated to produce 7% uncertainty for an overestimation/underestimation of the 90° pulse by 20°. Assuming this worst case scenario, the impact of an incorrect flip angle map on the reconstruction of $T_{1}$ from $T_{1}^{*}$ was calculated to account for less than 1%, under the experimental conditions described in the manuscript and considering the flip angle distribution shown in Figure 3B (mean ± s.d. = 4.8 ± 0.6°). The use of a preparatory 90° pulse is a fundamental requirement for the implementation of the proposed variable averaging method as, unlike in conventional inversion recovery, it allows the longitudinal magnetization to be nulled before proceeding with the train of small flip angle pulses. An incorrect saturation pulse can impact $T_{1}$ estimation even more severely, as it would not null the longitudinal magnetization. While the estimate of $M_{0}$ appears to be largely unaffected, simulations have shown that 10° uncertainty on the saturation pulse can lead to $T_{1}$ errors going from 16% for sample 1 to 40% for sample 14. Improved $B_{1}^{+}$ homogeneity, for instance by separating the transmit coil from the receive one or by employing adiabatic pulses, typically less sensitive to B_1_^+^ inhomogeneities, might mitigate this effect. While expected to be less of a problem for small angle pulses, an imperfect slice profile caused by truncation of the SINC function (i.e., finite side lobes) could be another reason for saturation pulses to differ from the expected 90°. Correction methods, normally based on look‐up tables, or the use of 3D acquisitions, are valid alternatives that can alleviate this problem.

### 5.2 Towards temperature mapping

To quantify temperature changes from $T_{1}$ variations, one requires to know the temperature sensitivity of the specific tissue or material investigated and to assign it to the appropriate region within the image through segmentation. In the presented work, a linear correlation could be established between $T_{1}$ and temperature (in the nominal range 25–60°C) in calibrated, doped‐water samples (c.f. Figure 5). Looking at previous studies at comparable (low) magnetic fields in general, a similar relationship ( $T_{1}$/temperature) was observed for a wide range of materials and selected tissue types.^31^, ^62^ Further extending this investigation, here the slopes k were found to be a linear function of $T_{1}$ measured for all samples at the chosen reference temperature (following Equation 9). This suggests that a tissue‐independent parameter K could be used to assess temperature changes as a sole function of $T_{1}$ measured at the reference temperature. This parameter would essentially be equivalent to the temperature sensitivity used in the PRFS method to convert the accumulated phase into temperature, which remains constant for nonadipose tissues.^63^ Although this prospect is highly promising, no study has yet examined the proposed dependence within tissue samples. Only very recently, a quadratic trend was detected in an agar‐based phantom at 1.5 and 3 T.^64^ Further investigation at different field regimes and with different samples will be needed to validate the proposed theory.

Overall, an average temperature accuracy of 96% ± 3% was calculated from the maps presented in this investigation, which corresponds to a mean underestimation by 1.5 ± 1.0°C. A temperature difference of 1°C from the ground truth is generally considered acceptable for hyperthermia treatments.^65^ While our results fall within the accuracy of the employed thermal camera, two main sources of uncertainty were identified for the proposed temperature estimation method. First, it is very important that the reference temperature used for the calculation of K is the same as the one chosen when performing the temperature mapping, which in a clinical scenario would be 37°C. Second, given the ratio $T_{1}^{b}/T_{1}^{a}$ (Equation 10), $T_{1}$ underestimation typical of LL sequences does not reflect on temperature (considering similar discrepancy); yet if the $T_{1}$ fluctuation around the expected value is not constant at the two temperature points, this uncertainty would heavily impact the estimated temperature by virtue of the small value of K. Maximizing $T_{1}$ mapping precision is therefore of the utmost importance. Here, an average $T_{1}$ precision of 7% ± 2% was observed throughout all samples and temperature points, leading to a measured average temperature dispersion within each vial (i.e., s.d.) of 3.0 ± 1.1°C, although a statistically significantly lower precision was achieved for the sample characterized by the lowest $T_{1}$. Because $T_{1}$‐based thermometry has so far been mainly employed as an alternative to PRFS in fatty tissue, a direct comparison between the presented results and the data available in the literature is challenging. An accuracy of 2.5°C and a precision of 2.7°C were reported in a porcine fat sample using the variable flip angle method and a 3‐T scanner.^35^ In a similar in vivo study performed with a 1.5‐T scanner, the temperature error ranged from 3.6 to 7.1°C in adipose tissue in human breast, with values as large as 28.6°C near fat/water and water/air interfaces.^36^ Despite the reduced SNR, the superior results achieved in this study represent promising ground for robust in vivo temperature quantification at low magnetic field. Existing results acquired in muscle and adipose porcine tissue samples at a similar magnetic field strength revealed a $T_{1}$ dependence on temperature in the range of ~4 to 5 ms/°C for a reference $T_{1}$ of 442 and 222 ms, respectively.^62^ Aiming for a 1°C error on the temperature estimate, the shallow slope sets an upper limit on the required level of $T_{1}$ precision, which is lower than the average of 7% measured here. Optimizing the sequence parameters for a specific range of $T_{1}$ values could improve SNR and lead to more uniform fitting outcomes within each vial. Noise reduction through higher performance hardware, advanced *k*‐space sampling strategies, and more elaborate reconstruction approaches (e.g., deep learning) would also be key to achieving further gains in precision.

### 5.3 Potential employment in mild hyperthermia treatment

Although $T_{1}$‐based thermometry has been used in thermal ablation as a complementary modality for temperature monitoring in fatty tissue, its employment in aqueous tissue may be suboptimal. While in the first case the linearity between $T_{1}$ and temperature is preserved, this is not the case in aqueous tissue past a specific thermal dose,^62^ mostly due to protein denaturation. Application‐wise, such a technique may therefore be better indicated for mild hyperthermia (39–43°C). A recent collection of guidelines for clinical mild hyperthermia treatments has recommended a minimum required temporal resolution of 20 s.^19^ This acquisition time is supposed to account for thermal washout due to the slow heating process^66^ and is at the moment only achievable through mapping techniques based on PRFS.^19^ Here, optimization of the presented variable averaging scheme could further shorten the total acquisition time, while extra gains could also be reached from a combination of strategies to enhance SNR, as described above (i.e., detection hardware, acquisition scheme, postprocessing). That said, the long exposure times typical of mild hyperthermia treatments, normally ranging from 30 to 90 min, may also enable relaxing the time constraints described.^19^, ^67^

The native spatial resolution used here was 180 mm^3^. While zero‐filling to achieve a reconstructed in‐plane voxel spacing of 1.5 mm may reduce partial volume effect artifacts and improve the localization of hot spots, the application of a Hamming window trades a higher SNR with a loss in spatial resolution. General recommendations have suggested a minimum requirement of 125 mm^3^, given the inherent difficulties of the currently available RF applicators to generate narrow energy distribution profiles.^19^ Although not far from the suggested limit, the larger dimensions employed in the reconstructed $T_{1}$ maps might not be sufficient for the correct identification of hot spots. The chosen voxel dimensions were dictated by the elongated shape of the vials and by the chosen imaging plane. While different voxel sizes were not investigated, optimization for specific body parts could theoretically improve the accuracy and precision of the reconstructed $T_{1}$ maps.

While low SNR and longer acquisition times are the unavoidable price to pay when working at low magnetic field, clinical applications are now in sight, thanks to innovative and creative solutions both in hardware and software. In addition, the technical advantages associated with the integration of therapeutic devices (e.g., a hyperthermia applicator and/or a linear accelerator for adjuvant radiotherapy) are not to be disregarded. Cross‐talk between the radio frequencies antennas conventionally used for hyperthermia^19^, ^68^ (which typically operate at 100 MHz^69^, ^70^, ^71^) and the working frequency of the MR system is expected to be reduced, hence limiting the need for additional filtering.^72^, ^73^ In light of the reduced Lorentz forces,^74^ a fully integrated low‐field MRI system for combined hyperthermia and radiotherapy could certainly be envisioned. Besides, the results presented here are expected to extend beyond the realm of low‐field MRI. Thermometry in adipose tissue represents a well‐known challenge for PRFS, because of the small temperature‐dependent chemical shift.^75^ Thanks to the increased SNR achievable using conventional 1.5‐T and 3‐T scanners, we believe that the proposed sequence could find employment in MR‐guided ablation of body parts rich in adipose tissue, such as breast and abdomen. A higher SNR and the possibility to use faster acquisition modes (e.g., echo‐planar imaging) can also be leveraged to further accelerate the proposed method, to achieve a fast and robust technique for both temperature and $T_{1}$ mapping. Overall, because the presented variable averaging scheme has been shown to partly compensate the bias in $T_{1}$ estimation typical of LL‐based approaches, we believe that this approach could be beneficial for a variety of additional applications that primarily rely on LL sampling (i.e., cardiovascular MRI) or for those applications that are inherently affected by low SNR (e.g., low gamma nuclei MRI).

## 6 CONCLUSION

This study presents $T_{1}$‐based MR thermometry as a credible alternative to the standard PRFS at low field (0.1 T). Despite the intrinsically longer acquisition times caused by lower sensitivity, creative approaches can be leveraged, such as the proposed variable averaging strategy. Besides, the presented method takes full advantage of the higher $T_{1}$ dispersion typically encountered at lower field regimes. Specific clinical MR‐guided procedures like hyperthermia could benefit from these developments, hence opening new territories for more accessible MRI applications. Thanks to the enhanced flexibility for multimodal technologies and exploiting the potential application of $T_{1}$‐based thermometry in fat‐rich body parts, low field could be a good candidate to explore hyperthermia in more challenging body parts. Ex vivo and in vivo investigations are inevitably required to assess the possibility of transferring the proposed MR thermometry modality into clinics.
